# Supplementary figures and images for: Evaluating the cleavage efficacy of CRISPR-Cas9 sgRNAs targeting ineffective regions of Arabidopsis thaliana genome
Source: PeerJ. 2021 May 21;9:e11409. doi: 10.7717/peerj.11409 (PMC8142926; doi:10.7717/peerj.11409)

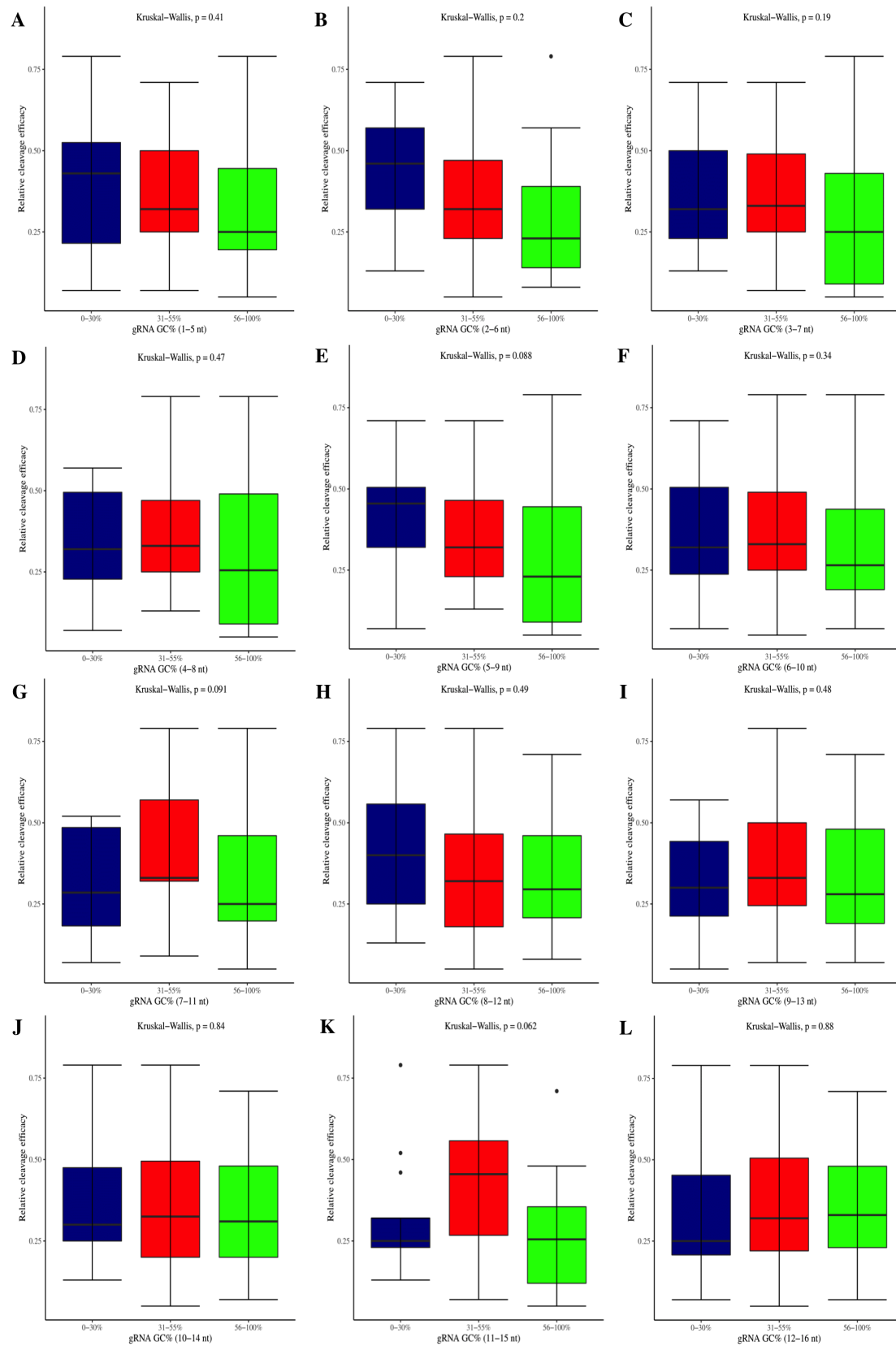

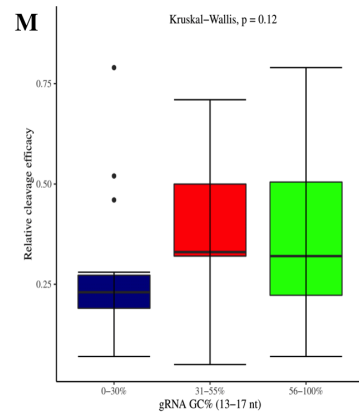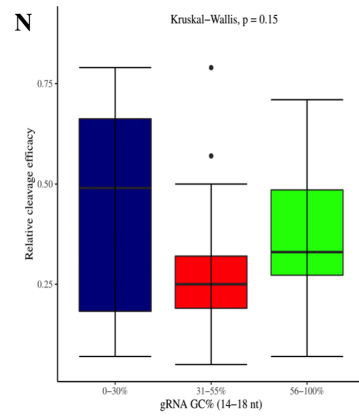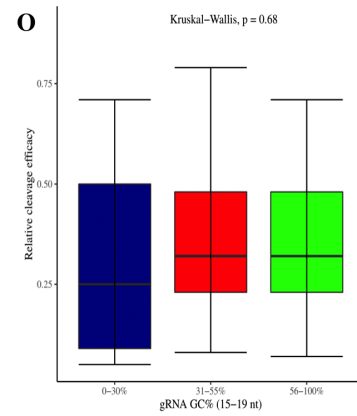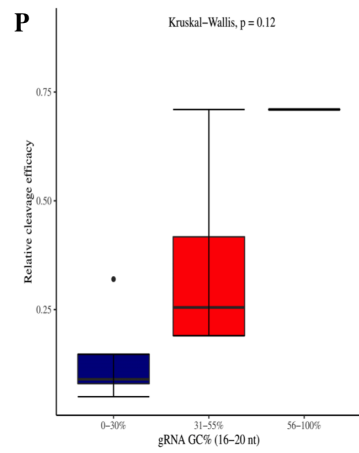

Supplement: Supplemental Information 2 — The graphical representation of analysis of GC content divided into three groups of ranges 0–30%, 31–55%, and 56–100% using a window size of 5 nt moving one nucleotide across the entire sequence of gRNAs. (A–P) No significant difference overall as indicated by the Kruskal-Wallis test. (A–I) Demonstrates a positive but non-significant increase in the cleavage efficacy for low and medium GC content groups (0–30% and 31–55%, respectively) up to position 13. (J–P) Describes a similar trend while moving away from PAM where the medium and high GC content groups (31–55% and 56–100%, respectively) impact the activity except for region 14-18 nt where low and high GC content groups (0–30% and 56–100%, respectively) are associated with increased cleavage efficacy (N). [file peerj-09-11409-s002.pdf]
